# Biomimetic Design and Assessment via Microenvironmental Testing: From Food Packaging Biomaterials to Implantable Medical Devices

**DOI:** 10.3390/biomimetics10060370

**Published:** 2025-06-05

**Authors:** Diana V. Portan, Athanasia Koliadima, John Kapolos, Leonard Azamfirei

**Affiliations:** 1 Department of Mechanical Engineering and Aeronautics, University of Patras, 26504 Patras, Greece; 2 Physical Chemistry Laboratory, Department of Chemistry, University of Patras, 26504 Patras, Greece; akoliadima@upatras.gr; 3 Department of Food Science and Technology, University of the Peloponnese, Antikalamos, 24100 Kalamata, Greece; i.kapolos@uop.gr; 4 Department of Anesthesiology and Intensive Care Medicine, George Emil Palade University of Medicine, Pharmacy, Science, and Technology of Targu Mures, 540139 Târgu Mureș, Romania; leonard.azamfirei@umfst.ro

**Keywords:** biocompatibility, biointegration, food packaging materials, implantable biomaterials, relevant biomaterial testing

## Abstract

Biomaterials and biomedical devices interact with the human body at different levels. At one end of the spectrum, medical devices in contact with tissue pose risks depending on whether they are deployed on the skin or implanted. On the other hand, food packaging and associated material technologies must also be biocompatible to prevent the transfer of harmful molecules and contamination of food, which could impact human health. These seemingly unlinked domains converge into a shared need for the elaboration of new laboratory evaluation protocols that consider recent advances in biomaterials and biodevices, coupled with increasing legal restrictions on the use of animal models. Here, we aim to select and prescribe physiologically relevant microenvironment conditions for biocompatibility testing of novel biomaterials and biodevices. Our discussion spans (1) the development of testing protocols according to material classes, (2) current legislation and standards, and (3) the preparation of biomimetic setups that replicate the microenvironment, with a focus on the multidisciplinary dimension of such studies. Testing spans several characterization domains, beginning with chemical properties, followed by mechanical integrity and, finally, biological response. Biomimetic testing conditions typically include temperature fluctuations, humidity, mechanical stress and loading, exposure to body fluids, and interaction with multifaceted biological systems.

## 1. Relevant Environments and Challenges in Bioproduct Assessment

The processing and integration of novel biomaterials and medical devices require rigorous testing before approval by the relevant body. This is a highly difficult task because of increasing complexity in structure and function, and is enabled through advances in materials science and engineering. Currently, several assessment steps involve animal models, with plans underway to restrict such activities. While U.S. applies increasingly stricter rules regarding animal welfare [1], the EU legislation sets a clear definite objective of eliminating the use of animals in scientific research and education [2]. Consequently, there is an important drive to shift health and environmental science testing towards in vitro systems that can at least in part reproduce natural/physiological conditions. In this context, the development of new experimental protocols and setups is in high demand for the investigation of biomaterials and devices in relevant environments.

In vitro biomimicry of the human body microenvironment, for example, involves the controlled reproduction of key physiological parameters such as temperature and dynamic conditions. Depending on the material type and device structure, specific standards, guidelines, and adapted protocols are needed. When looking at materials, testing of thermoplastics therefore requires assessment of their viscoelastic behavior and the potential for viscoplastic effects under mechanical loading at body temperature. Metallic materials must be corrosion-resistant to prevent ion release, which could trigger biochemical chain reactions in the body. Complex biomedical devices, such as biochips, require durable, non-toxic packaging and encapsulation to protect electronic components.

The operating environments differ depending on the biomaterial or the biomedical device purpose. Food packaging materials are subjected to various environmental conditions that can impact their integrity, safety, and effectiveness in preserving food quality. These conditions include temperature variations, from freezing conditions to high temperatures [3]; humidity and moisture, which degrade mechanical properties [4] and affect barrier properties [5]; light exposure leading to discoloration, oxidation, or loss of nutritional value [6]; mechanical stress resulting through pressure, impact, and friction during handling, shipping, and storage, which can lead to deformation [7]; and chemicals from cleaning agents, food acids, oils, or external pollutants that could alter their properties or cause contamination [8,9,10]. As a result, food packaging materials need to be biocompatible and capable of safeguarding food from contamination [11] and physical harm, while also preserving qualities like flavor, aroma, and texture. It is essential to test these materials to confirm they effectively fulfill their intended functions. Examples of testing steps are packaging migration and food contact, color bleeding, resistance to transpiration and perspiration, and foreseeable use assessment [12,13,14,15]. Furthermore, related to the interactions between food and packaging materials, it is admitted that the complexity of migration processes necessitates further research to develop globally recognized risk management strategies and standardized testing protocols [16]. Similarly to the food packaging materials, external or dermal biomedical devices should fulfill some of the above requirements.

The situation is extremely complex in the case of implantable medical devices made with combinations of materials and designed to perform multiple tasks. Implantable devices like pacemakers, sensors, and stimulators are widely employed for patient diagnosis, treatment, and health monitoring [17]. The environment they must perform in depends on the location within the body and is both complex and dynamic. Several key conditions and challenges must be considered to ensure biomaterial effectiveness and longevity: body temperature, which has a different effect on the materials compared to room temperature [18]; pH levels in normal and pathologic conditions [19]; exposure to physiologic body fluids such as blood, interstitial fluids, and sometimes cerebrospinal fluid (depending on location) [20]; mechanical loading like normal and shear stresses, and cyclic loading due to bodily movements (e.g., in joints, heart valves) [21]; wear [22] and fatigue [23] resistance under a high number of cyclic loads;, electrochemical corrosion, especially in metallic implants [24]; hydrolysis [25] and enzymatic degradation [26], which mainly affect polymers/thermoplastics; biocompatibility and immune response, like foreign body reaction leading to fibrosis [27]; inflammation, which affects implant function [28]; protein adsorption influencing cell adhesion and immune response; blood compatibility [29] to avoid thrombosis and hemolysis; and, last but not least, cellular interaction and integration, which refer to osseointegration [30] in the case of bone implants, endothelialization in vascular implants [31], or neural compatibility in brain implants [32] to maintain signal transmission.

To achieve a complete and efficient characterization of emergent materials and biomedical devices aimed at performing in one of the above environments, a holistic approach that combines several fields of science is needed [33]. While the foundational disciplines of medical science, materials science, and biology remain crucial for research and education in biomaterials science, emerging fields like mechanics, computer science, robotics, and nanotechnology are increasingly contributing to its advancement. Furthermore, the assessment of the performance of biomaterials and medical devices requires adaptation of experimental protocols depending on the material type, as well as the development of innovative testing machines to partially reproduce the specific operational environment. Human health benefits and safety are the targets in assessing the performance of these biomaterials and devices. Challenges are associated with the formation of multidisciplinary research groups and the creation of laboratory facilities. The use of multiple laboratories and their improvement through updated components is a solution. This requires, on the other hand, good organization and knowledge of all the steps to be implemented based on the research plan. Also, these studies need a back-and-forth approach that involves the gradual update and improvement in the testing procedures as the number of simultaneously tested parameters increases. More precisely, new, customized/homemade components may be required in a testing device to optimize the in vitro environmental reproduction of real operational conditions. The elaboration of bioreactors that allow the simultaneous investigation of biological and mechanical feedback of biomaterials and biomedical devices is an example. In biomedical bioreactors, mechanical loads can be applied to biological tissues during cultivation. However, the complex interplay between mechanical properties and cellular activity is highly influenced by the specific loading conditions, making it essential to replicate accurate physiological movements and forces within the bioreactor [34].

**Figure 1:**
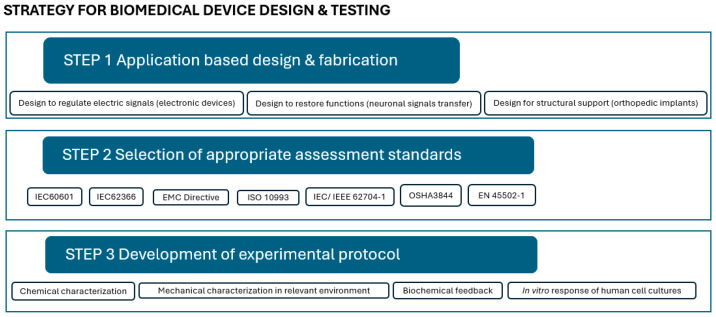
Schematic representation of the main steps to be followed in biomaterial assessment.

In the process from design to approval, three main steps, which are correlated, may be defined, as shown in Figure 1: (1) application-based design and fabrication; (2) selection of appropriate assessment standards; and (3) development of the experimental protocol.

Computational and analytical design and modeling have led to major breakthroughs in biomaterials and allowed the validation of theories. For instance, Portan et al. used semi-empirical modeling and software analysis [35] to predict and interpretate the response of primary osteoblast cells to various substrates and their characteristics. Deep learning enhances biomaterial functionality, with AI methods aiding novel design and machine learning predicting responses to environmental stimuli [36,37,38,39,40,41]. Although the validation of the computational models with real experiments is rarely performed, with these strong computational tools, the design of innovative biomaterials and medical devices can be undertaken according to a specific application (Figure 1, Step 1), while it is expected that upon providing the appropriate missing experimental protocols and tools, more researchers will combine computational tools with experimental results. In this context, one of the purposes of the present investigation is to clarify and describe a few missing laboratory protocols.

Design may be just for structural support, as in the case of orthopedic implants. In other cases, designs that confer low-power and high-speed efficiency are required, like in the case of devices for transmission or restoration of electrical signals [42]. The design is followed by the fabrication of the actual structure. The combination of various biomaterials produced by innovative technologies that are available nowadays allows the fabrication of efficient multifunctional structures capable of complex tasks in a target microenvironment [43]. Composite devices or materials made of multiple phases are most preferred since they enable multifunctionality [44]. The materials’ structure and their electrical conductivity, as well as other important features, foster the biointegration process [45,46]. This first step in the fabrication of novel biomedical devices is dedicated to defining key properties that will dominate the material/device in the relevant operational conditions. Thus, materials that replace hard tissues must be fatigue-resistant [47,48] to be load-bearing, the soft-tissue-replacing materials must be hydrophilic and highly biocompatible so that they facilitate tissue regeneration [49,50], materials for skin replacement need elasticity [51,52,53], dental materials must be thermostable [54,55,56], and electronic implantable devices have to be perfectly isolated by a coating [57,58,59].

The selection of the appropriate standards for assessment (Figure 1, Step 2) within a legal framework and for maximum safety is the next step. The FDA’s blue book memorandum #G95-1, a revised version of ISO 10993-1, “Guidance on Selection of Tests”, specifies that “in the selection of materials to be used in device manufacture, the first consideration should be fitness for purpose having regard to the characteristics and properties of the material, which include chemical, toxicological, physical, electrical, morphological, and mechanical properties”. This memorandum, which is a revised version of ISO 10993-1, governs the evaluation of medical devices [60]. In addition to ISO 10993, several standards are employed throughout different phases of characterization to investigate components of a biocompatible material or biomedical device. The sample’s shape, number, and testing conditions are defined in these standards. However, there are cases when standards are not specific enough and researchers need to establish their own protocols. While ISO standards play a crucial role in ensuring the safety and efficacy of biomaterials and biomedical devices, their broad and generalized nature often leads to challenges when addressing them for specific applications. For instance, ISO 10993 provides a framework for the biological evaluation of medical devices but may not address the unique requirements of emerging technologies such as nanomaterials or 3D-printed scaffolds. Moreover, standards like ISO 13485, which outlines quality management systems for medical devices, are not fully aligned with the ISO High-Level Structure (HLS). This misalignment can complicate integration with other management system standards, leading to implementation challenges [61]. Additionally, the absence of specific validation processes tailored to medical devices has delayed the adoption of new testing methodologies. Despite successful applications in other domains, there is hesitation regarding the predictive capacity of these alternative methods [62]. These examples underscore the need for more detailed and application-specific ISO standards to effectively address the diverse and evolving landscape of biomaterials and biomedical devices.

At this point, the development of new experimental protocols for complex and appropriate characterization and assessment correlated to the specific design and application is highly demanded (Figure 1, Step 3). Given the complexity of the body’s microenvironment, several factors must be taken into account: (i) atmospheric and bodily fluid-induced humidity, which affects radical processes in biomaterials, altering reaction pathways and kinetics; (ii) physiological conditions involving load, pressure, viscosity, and sliding speed, which generate friction and tribological phenomena, especially for internal devices; (iii) low-intensity electricity generated by the human body, with electrical potentials ranging from 10 to 60 mV at various sites; (iv) vibrations resulting from cyclic tissue loading in elastic structures; and (v) complex mechanical loading in the musculoskeletal system, as well as cumulative effects influencing biomaterial degradation, further accelerated by body temperature, which induces aging effects [63].

Consequently, each biomaterial/device must be tested depending on its nature, structure, overall characteristics, and type of interaction with the human body (external/internal/implantation site). The elaboration of experimental protocols must consider the use of traditional methods, as well as the construction of homemade setups that can closely mimic the microenvironment. Finally, biocompatibility and biointegration are the goals and refer to ensuring feasibility from all viewpoints: structural and mechanical resistance, and chemical and overall stability in a relevant environment, accompanied by biologic compatibility.

Compared to previous investigations, this paper highlights the importance of testing the materials in correlation to their operating environment, while describing in detail the major steps. It aims at bringing advancement for scientific knowledge in the field of biomaterials and food packaging product evaluation and testing, by offering step-by-step guidelines in (1) choosing innovative design criteria via multifunctionality and biointegration, (2) planning of the experimental setup using the optimum combination of traditional tools (standardized protocols and devices), and (3) applying efficient solutions for testing through methods beyond the state of the art, by employing adapted protocols for customized testing (incubators, bioreactors, or machines).

## 2. Biomimetic, Biointegration-Driven Engineering Design for Biomaterials and Biodevices

The engineering design of biomaterials and implantable devices involves numerous interconnected factors, such as dimensions, geometry, mechanical properties, and long-term compatibility with the surrounding microenvironment. While there is no single bear “optimal” design standard, implants can be optimized for strength, interface stability, and load distribution by selecting appropriate materials, surface treatments, and thread designs [64]. A cutting-edge approach to maximize the efficiency of biomaterials and devices goes beyond biocompatibility, and targets biointegration. To understand the concept, some terms must be clarified and defined:

- Biocompatibility is the commonly used term to indicate that a material or device accomplishes appropriate biological requirements. Biocompatibility has been described as the ability of the component in contact with, or implanted in, the human body, to enable an appropriate host response for a specific application. Biocompatibility or safety evaluation addresses the identification of an appropriate host response [65], which generally refers to non-toxicity and toleration. The biocompatible material/device retains one target function, and does not actively involve triggers that help the adaptation of the natural tissue to it. In this sense, a biocompatible material is considered inert and non-active, but neither is it toxic. Because of its biological inactivity, the biocompatible component may be considered an old generation technology.
- Biointegration goes beyond biocompatibility because it refers to an active and continuous cooperation of the biomaterial with the host body. Biointegration promotes cooperation of biomaterial and dermal or internal human tissues, and it involves functionalized materials like antimicrobial food packaging materials [66] or implants that enable the fast bonding of their surface with living tissues due to their engineered features. As previously stated, the new frontier in biomaterials design is based on the principle of biomimicry. This aspires to engineering biomimetic, bioinspired, and bioactive biomaterials that imitate the intricate extracellular matrix (ECM) composition and architecture while providing the necessary bioactive cues/instructive signals that determine positive interaction with the host, as well as control over cellular functions. Advancements in engineering, chemistry, biology, and medicine have been catalytic toward this goal [67]. Biointegration means ensuring feasibility from all viewpoints: optimum structural and mechanical resistance, chemical and overall stability in a relevant environment, and biologic compatibility.
- Biomaterial/biodevice functionalization seeks to improve the overall performance of the component. This means that multifunctionality is enabled. For example, in the case of food packaging materials, besides the main function of food protection, polymer functionalization allows grafting specific moieties and conjugate molecules that improve material performances [68]. In the case of biomedical materials, the target is cell differentiation [69] and formation of a new interphase area at the implant surface where the two initial components, synthetic and biological, work harmoniously together, leading to integration of the implant and the local tissue [35].

Achieving higher biointegration of biomaterials or medical devices is strongly influenced by their operating duration. This process requires careful design and programming of properties that cater to either short-term or long-term applications. For short-term use [70], biomaterials need to demonstrate adequate stability while being compatible with the biointegration process. Recent advancements in technology have enabled the development of 3D biomimetic and biodegradable materials that offer excellent performance for durations extending up to several months. These materials are designed to mimic the natural tissue environment, enhancing their integration with biological systems during this period. However, when considering long-term applications [71], maintaining the stability and mechanical performance of biomaterials becomes more complex. Long-term use presents significant challenges related to the degradation rate, as biomaterials must withstand prolonged mechanical stress and resist premature breakdown. Furthermore, the degradation process itself can lead to byproducts that may induce inflammatory responses or negatively impact surrounding tissues. To address these concerns, extensive research is being conducted on optimizing material properties, including improving their mechanical strength [72], enhancing degradation control, and ensuring minimal adverse biological responses over time. The stability and performance of these materials over extended periods are influenced by factors such as material composition, surface properties, and the interaction with the surrounding biological environment. Additionally, advances in nanotechnology [73] and surface engineering [74] are allowing better control over these properties, ensuring that biomaterials are tailored to meet the specific demands of their intended use, whether temporary or long-term. Ongoing studies focus on the design of advanced biomaterials that balance mechanical performance and degradation rates to improve human health outcomes and extend the functional life of biomaterials and biodevices.

**Figure 2:**
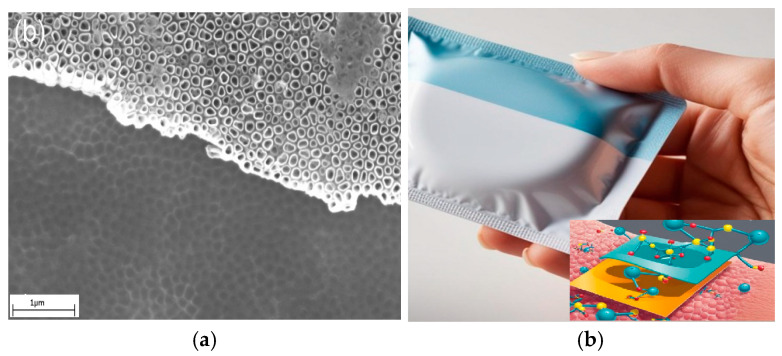

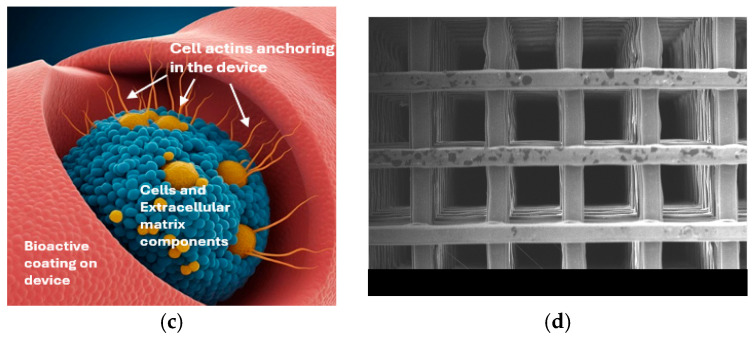
Suggestive SEM images from authors’ research or representation generated with Canvas: (**a**) SEM image of TiO_2_ nanotubes layer on titanium [76]; (**b**) thermoplastic food packaging; (**c**) human cells in contact with an implantable device; and (**d**) SEM image of a biodegradable polylactic acid scaffold.

The bibliography presents several reviews and case studies on strategies to improve the compatibility of biomaterials that are used in the human health sector, through a biointegrative approach. Various pathogens and microbes that affect health can potentially grow on food packaging materials, depending on several factors such as humidity, temperature, the type of packaging material, and whether the food inside is contaminated. Some of the common pathogens and microbes include bacteria (*Salmonella* spp., *Escherichia coli*, etc.), fungi (*Aspergillus* spp., *Cladosporium* spp., etc.) and viruses (Norovirus, Hepatitis A, etc.). To combat this problem, studies were conducted for the functionalization of food packaging materials towards antimicrobial resistance through surface tailoring like wettability adjustment, or incorporation of generally recognized as safe (GRAS) non-toxic substances—essential oils or natural acids. In the case of implantable materials, more complex methods have been used to optimize the biomaterials by conferring them biomimetic features that enable biorecognition processes in the natural tissue. Figure 2 presents an evolution of the biointegration approach.

The first attempts to functionalize biomaterials destined for implantation were towards coating the implant surfaces (Figure 2a). One well-known example is the orthopedic metallic plates’ functionalization by fabrication of nanostructures on their surface, such as TiO_2_ nanotubes on titanium [75,76]. Nanotubes’ role is to increase the contact surface area between the implants and the tissue, thus enhancing adhesion and at the same time ensuring antimicrobial properties. Later, incorporation of bioactive agents including antimicrobials into polymers is widely applied to improve food packaging materials’ performance and safety towards human health (Figure 2b). Antimicrobial packaging prevents microorganism growth while retaining its role as a barrier to water vapor and gases. This is achieved by incorporating antimicrobial agents into packaging via release, absorption, or immobilization. Some agents target metabolic or genetic pathways, while others alter microbial cell wall structures [77]. To overcome the challenge of creating stable interfaces with the rigid, planar components that make up medical devices, biointegrated wearable systems are being developed. Hydrogels and ionogels represent a class of stretchable active materials notable for their ability to closely replicate the properties of biological tissues. These systems utilize ionic mobility for conduction, similar to biological processes. Conductive hydrogels are used as coatings to intermediate the interface between the device and natural tissue (Figure 2c) [78]. Three-dimensional biodegradable scaffolds (Figure 2d) are regarded as the gold standard in biomimetic biomaterials, as they support cellular development and vascularization while gradually being resorbed. In “biohybrid” interfaces, a layer of living cells at the tissue–device interface not only better mimics native tissues but also serves as an active scaffold that promotes tissue regeneration, cell migration, and differentiation. Cell-populated scaffolds, composed entirely of biological components and living cells, aim for structural and functional integration between implants and host tissues [79].

The above studies prove the complexity of the up-to-date biomaterials and biomedical devices. To provide their complete characterization—structural, mechanical, chemical, and biological feedback—the overall characterization of these biomaterials in biomimetic environments needs to be performed. For each type of material, a complete set of experiments must be designed to obtain its performance profile in a relevant in vitro environment. This type of research requires correlating results from several scientific fields and a multidisciplinary investigation strategy. Below, the key testing procedures, devices, and protocols are described. The presented characterization tools and methods, as well as the strategy for the relevant microenvironmental testing, may be applied for both biodegradable and non-biodegradable biomaterials, with the specification that the latter need longer testing periods and, implicitly, greater resources.

## 3. Design of a Complex Characterization Strategy in Adequate Microenvironments

The following should be considered for relevance to the overall biological evaluation of a biomaterial/device: (a) the material(s) of manufacture; (b) intended additives, process contaminants, and residues; (c) leachable substances; (d) degradation products; (e) other components and their interactions in the final product; and (f) the properties and characteristics of the final product, according to ISO 10993-1. However, protocols for multiparameter testing in a complex biomimetic environment are not described. Due to its significance in biological evaluation, ISO Working Group 14 is creating a standard to define requirements for material composition, leachable substances, and the physical and biological characteristics of devices [60]. The food packaging materials must be compliant with the regulatory and environmental factors, food safety regulations, and sustainability concerns, including recyclability and biodegradability in different environmental conditions.

Although biocompatibility testing is associated with biological feedback several times, which includes testing with tools such as different types of cell cultures, contact with blood and enzymes, and other biological components, the selection of the biomaterial is made depending on its overall performance. The thermomechanical behavior of the biomaterial must be adequate so that it does not harm the natural tissue. Materials may be declared biocompatible upon cell culture testing; however, the challenge of operating in human body-like conditions, which involve an elevated temperature and constant loading in a humid environment, may further prove that they are not suitable for the specific application. The complex body conditions lead to a rapid and sometimes aggressive degradation rate, especially in the case of thermoplastic materials. For this reason, a complex thermomechanical characterization in combination with chemical testing is a priority. When the material is declared optimum from chemical and thermomechanical viewpoints, biological testing must be performed to achieve the overall characterization. This approach helps reduce costs and time. Minimizing “trial and error” experimentation can also be achieved by eliminating several candidate biomaterials based on chemical and mechanical characterization prior to biological testing.

For efficient experimental planning, two important aspects must be considered:

(1) The testing strategy may be top-down or bottom-up. The top-down testing strategy refers to the evaluation of a biomaterial or a biomedical device that is composed of more than one piece. Therefore, the research aims at testing the final product. In general, such a strategy is applicable if all the testing components inside the material/device are considered biocompatible on their own and the final target is the assessment of the final product in a relevant microenvironment. For complex devices, the testing is focused on the casing performance in terms of thermomechanical resistance and sealing, especially in a complex body-like environment. The experimental set depends on the nature of the external casing. Some biomimetic surface features may be added to the coating but, generally, the protective box is made of non-active biomaterials. On the other hand, the bottom-up strategy considers building the biomaterials and biodevices step-by-step; each component is functionalized through addition of key features that induce biorecognition and fasten biointegration. For example, electrolytes must be both conductive and non-toxic. Each component can be improved with a feature that makes it biocompatible or bioactive: antimicrobial effect, flexibility to match human tissue’s structure and consistency, together with boosted overall performance in a body-like environment. The bottom-up approach is used especially in the case of biodegradable composite biomaterials that imply the elimination of each component in the body. All the components must be non-toxic and active whenever possible, and must reduce the immune response. Drug delivery systems are also incorporated into the biomaterial to make it more effective [80].
(2) Short- vs. long-term implantation is a critical aspect that is decisive regarding the experimental setup conditions and duration. Until now, most studies have presented biomaterial testing protocols at room temperature. Lately, the degradation of biomaterials in simulated body fluids like, for example, Hank’s balanced salt solution (HBSS), was investigated. The experiments had the scope to evaluate the properties’ degradation in the case of non-biodegradable materials, and the degradation rate until complete dissolution regarding biodegradable biomaterials. While the degradation of resorbable materials is studied for up to one year, for other biomaterials this period may be extended for as long as necessary, even for some years, and is usually performed in vivo [81]. Degradation refers to several properties associated with chemistry, such as, for instance, corrosion or the mechanical behavior of biomaterials, like elasticity modulus.

The planning of the experimental setup depends on the biomaterial type and structure. The proposed biomimetic testing protocols are designed to address the temporal dynamics of biointegration—such as differences between short-term and long-term implant performance—by allowing natural biological processes to guide the research framework. Specifically, once the characterization tools and methods described in this section are established, a strategy for monitoring the biointegration of the biomedical material or device can be developed. This strategy should follow a timeline aligned with the natural sequence of events occurring in the biological microenvironment (see Table 1). Therefore, careful planning is essential prior to initiating the research. This process requires a convergence of engineering and biological tools, and monitoring should be carried out through dynamic simulations that replicate real-life physiological conditions.

Below, the main traditional and novel biomaterial categories are presented with regards to their corresponding key properties that need to be thoroughly investigated. A description of the characterization trends for different biomaterial categories and biomedical devices is provided, together with useful laboratory observations.

### 3.1. Characterization Based on Material Classes

The key aspects in the characterization of the most common traditional (metallic, silicones), modern (carbon-based materials, coatings), and innovative (3D materials—scaffolds and hydrogels) biomaterials are described below, based on specific behavioral trends in their relevant environment.

#### 3.1.1. Metallic Biomaterials

Metallic biomaterials are applied in orthopedics and imaging. Titanium and its alloys are the leading bio-inert metals in biomedical engineering due to their machinability, strength, biocompatibility, and corrosion resistance—traits that make them critical for durable and effective medical treatments [82].

Less popular alloys like surgical stainless steel (316L) and cobalt-chromium (CoCr) alloys were used previously for fracture fixation, angioplasty, and bone remodeling. However, some issues were observed in stainless steel related to its corrosion and biocompatibility, nickel and chromium allergies, brittleness, limited flexibility, potential for inflammation and infection, and MRI interference [83,84,85]. Cobalt chromium also presents significant drawbacks, especially related to corrosion, ion release, biological compatibility, mechanical performance, and potential long-term adverse effects on surrounding tissues [86,87]. Though known for low corrosion, wear, and friction, these materials may degrade in harsh microenvironments, releasing metal ions that cause local inflammation, osteolysis, and systemic issues like hypersensitivity. Osteolysis can destabilize implants, leading to failure, revision surgery, or complications [88].

The appropriate setup for the investigation of biomedical metals in a relevant environment must combine experiments of cyclic voltammetry in a simulated body fluid for different time periods followed by the mechanical testing of the samples after immersion, under electrochemical conditions. The feedback of the metal to an electrochemical environment like the body will offer information about the rate of ion release due to surface reactivity. The surface degradation impacts the mechanical performance of metal, especially when the material is subjected to the electrochemical microenvironment for a prolonged period. Therefore, a combination of surface and mechanical characterization after exposure of the metallic samples to cyclic voltammetry through immersion in a simulated body fluid for prolonged time periods up to months is recommended.

#### 3.1.2. Carbon-Based Materials

Carbon-based nanomaterials are promising for biomedical uses like drug delivery and biosensing, thanks to their unique thermal, mechanical, electrical, and optical properties. Their hybrid inorganic–organic traits support biomolecular interaction and light responsiveness. Chemical modifications help mitigate toxicity for safe biological use [89]. However, the toxic effect of carbon-based biomaterials is still questioned.

Carbon biomaterials are categorized by (1) dimension—from 0D nanostructures (e.g., carbon dots, fullerenes) to 3D forms (e.g., graphite, diamond); (2) crystallinity—from crystalline (diamond, graphite) to amorphous (pyrolytic carbon); and (3) carbon hybridization—from sp^2^ (graphite) to sp^3^ (diamond) [90]. While the excellent mechanical performance of carbon-based biomaterials is well known, the focus when characterizing these materials must be towards their chemical and structural composition. A variety of analytical techniques can be used to investigate the structural and chemical properties of carbon-based materials, including carbon nanotubes, graphene, activated carbon, and biochar. X-ray diffraction (XRD), optical microscopy, elemental analysis, scanning electron microscopy (SEM), transmission electron microscopy (TEM), field emission scanning electron microscopy (FE-SEM), high-performance liquid chromatography (HPLC), and inverse gas chromatography (IGC) are a few of these [91].

When planning microenvironment testing, it is crucial to assess both the electrical conductivity and mechanical response of these materials after prolonged exposure to simulated body fluids over varying time periods. From a toxicological perspective, evaluating the response of human cell cultures to a culture medium containing different concentrations of a carbon-based material will offer insights into its biological feasibility. Additionally, these tests should be complemented by chemical analysis of the simulated body fluid after the carbon material has been immersed to monitor any local release of carbon species.

#### 3.1.3. Coatings

Surface characteristics are a critical determinant of an implanted biomaterial or device. Therefore, the chemical and morphological properties of the biomaterial surface are highly valued as they can enhance its mechanical stability, biocompatibility, and the ability of cells to migrate, proliferate, and differentiate, followed by endogenous tissue ingrowth. Coatings are created or applied to the surface of a biomaterial to give it new, healing-stimulating properties [92]. Coatings may be fabricated from the material itself, as in the case of titanium; the titanium surface can be nanostructured, in a nanoscale range, through electrochemical processes [75,76]. This nanoscale titanium oxide layer plays an important role for the protection of the metal against corrosion. Other methods like the addition of polymer coatings on implants are applied for functionalization. This solution mainly addresses resistance and biological issues, including antimicrobial properties, biomimetic features that enable tissue biorecognition, and protection of the natural tissue from the possibly harmful and toxic components at the interior of a biodevice.

Because coatings are thin films, in vitro testing under body-like conditions—to observe the coating stability and the chemical risk when in contact with the simulated body fluid—is mandatory. Surface characterization of the coatings through imaging and chemical techniques is needed. In addition, chemical analysis to monitor the local release of ions or macromolecules in the simulated body fluid is required. The coating’s main weakness is caused by the combination of temperature, humidity in the complex body fluid, friction, and, in some cases, like orthopedic implants, complex mechanical loading.

The tribological behavior and friction resistance of coatings is an essential aspect in the assessment of their performance in enhancing the durability of components, especially in high-stress environments like in the case of biomedical implants [93]. The efficient characterization of a coating must be made considering its microstructure, chemical composition, microhardness, and tribological changes upon immersion in physiological body fluids.

#### 3.1.4. Thermoplastics and 3D Scaffolds

One important feature of thermoplastics in biomedical applications is their processability. The thermoplastic is a viscoelastic solid at room temperature. It consists of long linear chain molecules that, above the softening temperature, exhibit large-scale chain mobility and deformation due to shear forces. This modification is reversible. The thermal motions of the chain segments are enough to overcome intra- and intermolecular forces above this temperature. The behavior of thermoplastics is influenced by the types of additive, chain morphology, structure, and crystallinity [94]. Because of their strongly temperature-dependent behavior, thermoplastics for biomedicine must be thoroughly investigated with respect to their performance at body temperature under aging and cyclic conditions. Thermomechanical analysis is applied to observe their transition temperature from glass (crystal) to a rubbery state and the threshold where their thermoelastic behavior becomes thermoplastic. More precisely, the degradation of their properties is not significant when performed in the body microenvironment for some weeks, but will suddenly accelerate and end dramatically in a very short time after that threshold is overcome. In the case of food packaging materials, the temperature oscillations from freezing conditions to room temperature cause damage.

The degradation rate and the property degradation of biodegradable thermoplastics must be investigated after immersion in physiological fluids for several months. Dynamic Mechanical Analysis (DMA) to understand the viscoelastic and dynamic response of the material under thermal and dynamic loading in a relevant environment is also important. Finally, the degradation of the thermoplastic structure and its overall property variation due to macromolecule denaturation because of exposure to a dynamic environment are important. In addition to this, depending on the specific application, thermoplastics must be mechanically characterized (tensile, compression, low energy impact) after prolonged immersion in simulated body fluid at body temperature. In the case of 3D scaffolds, their weight variation upon immersion in simulated body fluid is investigated in parallel with the degradation of their mechanical properties.

Although the possibilities of several thermoplastics are extensive due to their biocompatibility, any potential structural application is limited by their weak mechanical properties. Generally, the improvement in mechanical properties implies the sacrifice of biocompatibility. For this reason, several solutions are explored in the direction of thermoplastic-based composite biomaterials; the reinforcements vary from titanium oxide powders to carbon-based materials.

#### 3.1.5. Silicone-Based Biomaterials

Silicone is a synthetic polymer widely used in the biomedical industry as an implantable device owing to its excellent mechanical properties. Although referred to as biocompatible, silicone has an inert nature, which makes it non-active. With a water contact angle of 101° to 109°, silicone’s strong flexibility and excellent hydrophobicity are some of its essential physical characteristics. However, when silicones come into contact with host tissue they are vulnerable to microbial contamination. Antibacterial properties are needed, much like in the case of medicinal products. However, silicone’s ability to retain its mechanical qualities between −40 °C and +185 °C is a huge benefit, particularly regarding implantation in the body [95]. For this reason, research is directed mainly towards biointegration, surface modification, and improvement strategies. In this case, the imaging of the surface morphology and the investigation of its properties like contact angle, nano-stiffness, and roughness are targeted after functionalization through methods such as surface coating, physical or chemical modifications, and treatment with antibiotics or plasma-activated surfaces to develop the resistance to bacterial infection.

The complete set of relevant tests of a silicone biomaterial requires characterization before and after maintenance in a relevant environment. Also, biological tests by monitoring the response of cell cultures to the material, as well as of antimicrobial properties, need to be performed. An in vivo investigation that was conducted a long time ago showed that free silicone migrates from the implants to adjacent tissues and distant sites, such as the spleen or liver, and is chemically modified [96]. This makes the aging environment crucial for adequate testing. Since animal models are not encouraged, the testing conditions must be adjusted in an in vitro environment that closely mimics the real one. Chemical characterization of silicone and the simulated body fluid after their long-term interaction is going to help in understanding the influence of one component on another and the possible consequences on the host tissue.

#### 3.1.6. Biomimetic 3D Hydrogels

Because of their high equilibrium water content and exceptional biocompatibility, as well as biomimetic features similar to those of human tissue, hydrogels are among the most promising groups of biomaterials for biomedical applications [97]. However, the characterization of hydrogels to assess their performance, failure, and lifetime raises concerns because of their evident fragility. Chemical and physicomechanical tests have been conducted on hydrogels to address these problems. Understanding the morphological and microstructural characteristics of these materials is crucial since they are challenging to work with under melt or solvent settings [98].

Mechanical and surface characterization of hydrogels involve evaluating their rheological behavior, stretchability, and lubrication properties. Rheological testing at both macro and micro scales is critical for understanding the mechanical characteristics of all types of hydrogels, particularly supramolecular hydrogels, which exhibit enhanced shear-thinning and self-healing due to their self-assembled, non-covalent structures. Techniques such as micro rheology, dynamic light scattering, and tribological analysis are commonly used for such assessments. Unlike covalently crosslinked hydrogels, those formed through non-covalent interactions tend to show greater stress relaxation. Although the weak bonding in supramolecular hydrogels can limit mechanical strength, integrating them into multi-component interpenetrating network (IPN) hydrogels can improve tensile performance and enable structural recovery after deformation [99].

Due to their structural characteristics, testing hydrogels in physiologically relevant environments is essential. Aside from a few experiments—such as micro-tensile testing of the dry phase—most tests are conducted in simulated body fluid (SBF). Enhancing the testing setup by maintaining the system at body temperature accelerates the hydrogel’s degradation rate, providing more realistic insights into performance. Additionally, chemical analysis of the SBF after hydrogel immersion can reveal potential alterations in physiological parameters, indicating interactions or leaching of degradation products.

### 3.2. Overall Characterization: Surface, Chemical, Mechanical, and Biological Behaviour

The broad range of characteristics, properties, processes, and phenomena that need to be investigated in the case of innovative biomaterials and biodevices is not described in a single standard document. Therefore, the scientific community must find its own protocols and procedures to ensure complete assessment and safety in application. Although the lack of specific rules and the detailed description may cause confusion, the advantage is that the scientist may apply his own holistic approach for bedside transition of a novel biomedical product. The multidisciplinary dimension of such research is the most important step in achieving this goal. Since ISO/ASTM and other standards are not yet unified to offer the necessary support in this direction, groups with expertise in different scientific fields and with a background in laboratory research can elaborate their own guidelines, which is also the purpose of the present research. The sections below concisely present some of the most important experimental tasks for the characterization of biomedical materials and devices, together with key laboratory aspects and technical solutions for the handling and analysis of the samples.

#### 3.2.1. Surface Characterization

Surface imaging

The initial surface analysis is made on the control samples that have not been subjected to functionalization or exposed to an aggressive environment. The subsequent investigation of the samples is performed after the application of surface modification. Surface characterization may be divided into two main types, imaging and property investigation. Imaging is extremely important for the monitoring of the functionalization, as well as of the effects of the microenvironment on the integrity of the material. The simplest imaging laboratory tool is the optical microscope. However, this can be used only in the case of transparent specimens like thermoplastic coatings and its magnitude power is too low for the analysis of nanomaterials. The most used imaging techniques for the analysis of biomaterials are scanning electron microscopy (SEM) and transmission electron microscopy (TEM), which use a focused beam of high-energy electrons to scan the surface of a specimen or a high-energy electron beam that is transmitted through an ultra-thin specimen, respectively. SEM is used for millimeter to centimeter lengths/widths and millimeter-thick specimens, and it allows analysis of the surface structure/geometry. The exact required dimensions of the specimen depend on the microscope model. TEM, on the other hand, is used to analyze powder or liquids.

A few important aspects must be considered in the handling of the specimens that need to be addressed, depending on the material nature. The orthopedic metallic materials present particularities related to their cutting procedure. To obtain samples with smooth margins, laser technology or water jet technologies must be used for their cutting. The advantage of metals in relation to SEM is that they are semiconductors, and therefore can be observed without gold sputtering. This is not the case for non-conductive materials like thermoplastics. Most of the specimens used for SEM analysis are sacrificed, as they cannot be recovered after gold sputtering; for expensive materials, the cost of the specimens must be considered. An adjustment of the number of specimens can be made to a certain extent by reducing it for the specific imaging analysis. However, at least one specimen must be calculated for each measurement (i.e., specimens after 1, 3, and 7 days of immersion in the simulated body fluid). In this way, the surface structure and the degradation of a property may be crosschecked to confirm theories.

**Figure 3:**
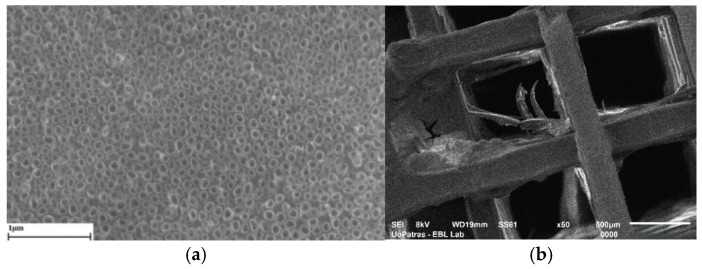
SEM images of biomedical materials: (**a**) titanium dioxide nanotubes [100] and (**b**) polylactic acid scaffold after immersion in simulated body fluid (unpublished work).

Another challenge in handling specimens is related to very thin materials with electrostatic behavior, like, for example, ultra-thin films produced by electrospinning or carbon-based films. These types of specimen can be removed by mistake even by the laminar air flow and can hardly be fixed in one place. To avoid specimens’ loss, ionizing air blowers for active neutralization, an electrostatic discharge gun, or other antistatic solutions must be applied. Figure 3 shows SEM images of two specimens investigated for biomedical research.

Figure 3a is a nanoscale magnitude image of a TiO_2_ nanotube layer that has been synthesized on biomedical titanium to protect it from corrosion. The efficiency of functionalizing these modified metallic surfaces may be evaluated by subjecting them to long-term cyclic voltammetry and observing them regularly by imaging to calculate the rate of surface degradation, ranging from pit formation to layer removal. The nanotubular structure can be well observed in the SEM photo and offers valuable information on the geometry, homogeneity, and dimension of the fabricated nano layer. In the image in Figure 3b, the SEM image of a polylactic acid 3D scaffold may be observed. The scaffold was exposed to a simulated body fluid for 21 days at 37 °C under rotational dynamic conditions. It may be seen that its degradation process starts with exfoliation and plasticization of polylactic acid fiber. In this case, the SEM micrograph provides information beyond geometry and dimensions, indicating possible degradation mechanisms involved in the process. The phenomena of structure degradation mechanisms in thermoplastics due to macromolecule release, because of exposure to a dynamic environment involving an elevated temperature, flow of a physiological fluid, and small compression loading or other bioreactor conditions, becomes more evident after imaging of the samples.

Surface properties

Other key surface characteristics that intermediate and contribute tremendously to the biointegration of the biomaterial are the wettability (hydrophobicity/hydrophilicity), the surface roughness, the stiffness/hardness measured by nanoindentation, and the tribology/wear properties. Surface characteristics are important for food packaging materials, and they are tailored to reduce microbial activity, as well as to ensure comfort when in contact with human skin. Regarding biomaterials that operate in the human body, it is crucial to understand the effect of the loss of macromolecules through removal due to the aging condition, which is, as previously mentioned, a cumulus of conditions in the human body microenvironment—temperature, friction, compression, tensile loading, biochemical degradation, etc. This is studied initially by observing the degradation of the surface properties. In the case of biomedical metals, the ideal relevant test to study the degradation of their surface under adequate biomimetic conditions is to apply cyclic voltammetry in simulated body fluids over extended time periods, and further by regularly measuring the mentioned surface properties (wettability, roughness, and hardness).

Biomaterials must not provoke any adverse reactions upon contact with biological tissues. One key factor affecting this compatibility is surface wettability, which is typically assessed by measuring the contact angle. Wettability refers to how readily a liquid spreads on or adheres to a solid surface, and is evaluated through contact angle measurements. This property is influenced by three main forces: the surface tension of the solid, the surface tension of the liquid, and the interfacial tension between them. In biomedical applications, adjusting the wettability of a material is applied to control its antimicrobial effect. Also, biomaterials are commonly employed in modern medicine as blood-contacting devices, such as stents, prosthetic heart valves, vascular grafts, hemodialysis membranes, vena cava filters, and blood bags. For these types of applications, wettability is extremely important. When blood meets these materials, platelets often adhere to the surface, depending on its wettability, and become activated. This activation triggers the release of prothrombotic factors, initiating the clotting process. Under normal physiological conditions, thrombosis and complement activation serve as protective mechanisms to prevent blood loss. However, when triggered by foreign materials like medical devices, these responses become undesirable, leading to the formation of blood clots and reduced biocompatibility. Therefore, a truly biocompatible blood-contacting device is one that does not induce thrombosis [101].

The substrate topography and stiffness are key material properties for biomimetic tissue engineering because they enable the appropriate biointegration by triggering a positive response of human cells to substrates. Properties such as texture, scaling, roughness, surface energy (wettability), and hardness affect the development, morphology, and adaptation of cells, and, consequently, the biointegration in the host body. Roughness is measured by atomic force microscopy (AFM), which works by scanning a sharp probe over a material’s surface to create a high-resolution 3D topographic map. As the tip interacts with surface features, it causes deflections in the cantilever, which are detected by a laser and photodetector system. These deflections correspond to height variations on the surface. From the generated data, roughness parameters such as average roughness (R_a_), root mean square roughness (R_q_), and maximum height (R_z_) are calculated. AFM offers nanometer-scale precision and is a non-destructive technique suitable for use in air, vacuum, or liquid environments. Nanoindentation is usually installed in the same device as AFM, and measures stiffness and roughness by pressing a sharp indenter into a material’s surface and analyzing the load–displacement response to determine mechanical and surface properties at the nanoscale [102].

The tribological performance of materials has been extensively investigated using various methods, such as ball-on-disc, ball-cratering, fretting, and rubber-wheel abrasion tests. An essential consideration in selecting a suitable tribological test model is the type of mechanical stress that the material system is expected to endure in practical applications [103]. Given the ongoing demand for improved implant designs and novel materials, substantial effort must be dedicated to in vitro wear assessment—both for evaluating new materials and designs, and for analyzing wear in explanted implants. Early-stage in vitro testing typically begins with standardized pin-on-plate experiments, which help rank material combinations and eliminate unsuitable options early in the development process [104].

#### 3.2.2. Chemical Characterization

Setup preparation

**Figure 4:**
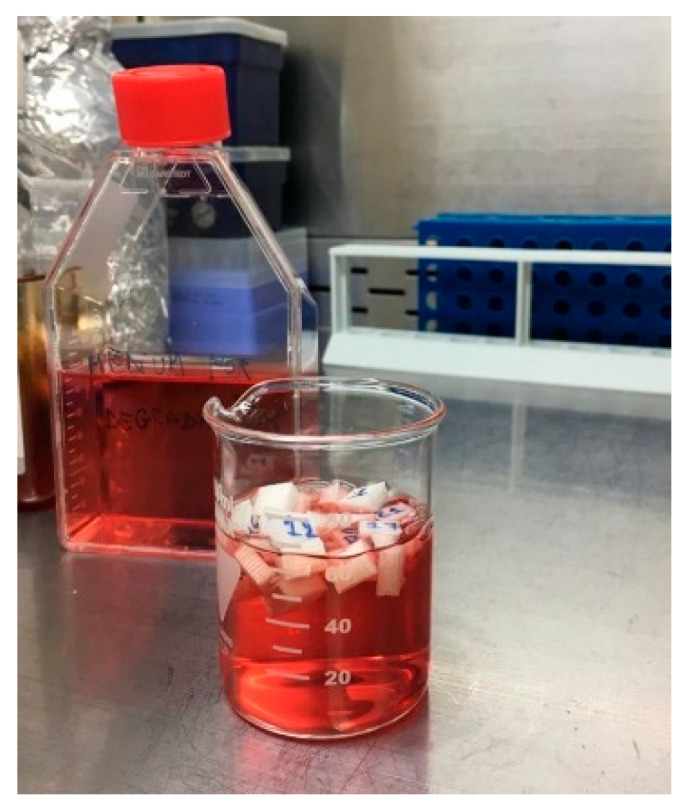
Three-dimensional scaffolds immersed in cell culture medium. Figure 4 is a simple laboratory setup that allows the investigation of a biomaterials’ degradation in a simulated body fluid, as well as their interaction. The fluid represents the cell culture medium, which contains blood cells, enzymes, proteins, and other biochemical components. The biomaterial suspended in the fluid is a 3D thermoplastic scaffold. Their interactions result in the release of macromolecules from the biomaterial in the cell culture medium and the absorption of biochemicals from the cell culture medium in the scaffold material.

Chemical analysis of biomaterials is used to determine their composition after fabrication and functionalization. However, the traditional methods are applied to the materials themselves and are not used for the analysis of the interactions with a relevant microenvironment, which is a critical step in obtaining a clear understanding of how the material–fluid–tissue impacts each component separately. For this system analysis, new protocols are suggested (Figure 4).

Material characterization before and after immersion in simulated body fluid

The chemical characterization of biomaterials begins with the analysis of a control sample that has not been exposed to any treatment or modification. The most accessible characterization method for solid samples, which can be performed at the same time as the imaging analysis, is energy dispersive X-ray spectroscopy (EDX), which identifies the material’s phase composition and structure, giving the exact chemical elements that comprise the sample and their concentration. For more advanced analysis, X-ray diffraction (XRD) provides information on the chemical compounds/complexes, the crystal structure, the phase composition, and the lattice composition [105]. A disadvantage is that the two techniques cannot directly analyze liquid samples. This is technically possible with EDX but there is a need for special sample preparation, like freeze-drying, drop-casting onto a substrate, or using sealed environmental chambers. Standard XRD also cannot analyze typical liquids, but specialized, less accessible techniques (i.e., synchrotron-based liquid XRD) can study the molecular structure or short-range order in liquids. EDX may be used on samples up to 1–2 cm in diameter, while XRD permits the analysis of a few milligrams of finely ground powder and solid samples up to 2–3 cm wide and a few mm thin. Some XRD instruments can handle larger samples, but uniform thickness and flatness are key for good results. EDX may analyze flat and rough surfaces, while XRD works for flat and smooth surfaces to ensure proper diffraction. Finally, important technical details of these techniques are as follows: (i) SEM-EDX must be vacuum-compatible and conductive, (ii) TEM-EDX must be electron-transparent, so it requires very thin sections, and (iii) XRD requires a flat surface. Helpful methods to adjust the samples for these techniques involve:

- Trimming down samples to adjust dimensions;
- Coating with gold, platinum, or carbon (~5–20 nm thick) using a sputter coater to make solid samples conductive;
- The use of double-sided carbon tape to fix powders;
- Polishing to make the surfaces for analysis flat and smooth.

XRD is applied in the case of metals, ceramics, minerals, or crystalline solids. Organic materials, polymers, and biochemical samples are analyzed by Fourier transform infrared spectroscopy (FTIR), which works for amorphous materials too. FTIR indicates the types of chemical bonds, the functional groups, the molecular structure, the degree of purity or contamination, polymer characterization, or changes in chemical composition (before/after reaction or aging). Consequently, FTIR is an appropriate method to analyze polymeric biomaterials before and after immersion in simulated body fluid.

Another well-known tool is nuclear magnetic resonance (NMR) spectroscopy, which can be used to analyze the chemical structure of polymeric macromolecules, identifying the different types of monomers, their linkages, and even the molecular arrangement. Solution NMR is ideal for polymeric systems dispersed in solvents. Raman spectroscopy can be additionally used to study vibrational modes of polymeric bonds. At the same time, X-ray photoelectron spectroscopy (XPS) is becoming more and more popular as it is a highly effective surface-sensitive analytical technique that is used to study the surface chemistry of biomaterials. It is especially employed for examining surface modifications, biomaterial–protein interactions, surface oxidation, and corrosion. Compared to other methods, XPS shows how elements are bonded, the functional groups, and crosslinking. It helps in analyzing the success of a surface treatment or the degree of immobilization of a bioactive molecule on a surface. Its most critical application to biomaterials is to detect adsorbed proteins from biological fluids (e.g., serum, plasma), which is extremely useful in the case of thermoplastic scaffolds’ immersion in simulated body fluid, to analyze the surface chemistry for osseointegration or immune response in implants (e.g., titanium, polymers), verify surface coatings or loaded molecules in drug delivery systems, and assess crosslinking and surface-bound molecules in hydrogels [106].

Fluid choice and characterization

Simulated body fluids (SBFs) are used in the laboratory to investigate biomaterial interaction with biomimetic fluids like body fluids. This interaction usually involves deposition of salts and other biochemical components from the fluid on the biomaterial surface, corrosion, or biodegradation. The classic and most common simulated body fluids are standard SBF, Hank’s balanced salt solution, Phosphate-Buffered Saline (PBS), or Ringer’s Solution. These solutions are primarily composed of inorganic salts dissolved in water, which mimic the ionic composition of various body fluids (like plasma or extracellular fluid), and in some cases, maintain pH balance and osmolarity like physiological conditions. The disadvantage of these solutions when studying biomaterials is that they do not really mimic the complexity and priority of events that take place at the implant–tissue interphase. The most important components that meet the biomaterial of the biodevice in a real situation are the blood and plasma components: cells, enzymes, proteins, and other biochemical complexes. When a biomaterial is implanted in the body, a cascade of biological events unfolds at the implantation site. These events are part of the foreign body response and happen whether the implant is metallic, ceramic, polymeric, or composite. The most important biological events are presented in Section 3.2.4.

A challenge associated with a more adequate in vitro study of biomaterials consists in designing an investigation in the presence of cell culture medium, because this is significantly more complex and nutrient-rich than simulated body fluids, and it is designed to support cell growth, survival, and function, rather than just mimic ionic conditions. Compared to simulated body fluids, the cell culture media contain amino acids, vitamins, glucose, salts, pH buffers, proteins, and growth factors; therefore, they mimic interstitial fluid more closely and permit the execution of functional studies. In vitro, it is ideal to maintain the biomaterial in a cell culture medium at 37 °C. Another important aspect is to constantly adjust the analogy between liquid and biomaterial by evaporation control for comparable results in long-term studies. The chemical analysis of the biomaterial at regular time intervals after immersion can be performed with the techniques described above. For the analysis of polymeric macromolecules dispersed in a liquid, NMR can be used as an accessible technique. Other solutions are dynamic light scattering (DLS), which measures the size distribution of polymer particles or macromolecules in solution by analyzing the fluctuation of scattered light caused by Brownian motion, and can provide the hydrodynamic radius of the polymer and give insights into polymer size and polydispersity; gel permeation chromatography (GPC), which separates polymer chains based on their size (molecular weight) in solution and provides information about the molecular weight distribution and polydispersity index (PDI) of polymer macromolecules; and light scattering (static light scattering, SLS) in conjunction with DLS for determining molecular weight and the size of polymers in solution. Crystallin powders in cell culture medium can be investigated by XRD, FTIR, RAMAN, or XPS, previously described above.

Finally, metallic biomaterials must be thoroughly investigated to determine the potential release of ions in the body. The release of high ion concentrations may lead to local toxic effects and an inflammatory response or worse, and enter systemic circulation, potentially affecting distant organs. The characterization devices for metallic ions in a fluid are inductively coupled plasma–optical emission spectroscopy (ICP-OES) for very sensitive elemental analysis of metal ions in solution [107]; atomic adsorption spectroscopy (AAS), which measures the concentration of specific metal ions; UV-Vis, which can detect transition metal ions or metal complexes; XPS, although only if the analysis is for dried residues; or FTIR if the metal forms part of a coordination complex with organic ligands [108].

#### 3.2.3. Static Mechanical and Thermomechanical Characterization

Ensuring the thermomechanical performance of biomaterials and biomedical devices remains one of the most complex and demanding challenges in the development of novel implantable components. This difficulty arises from the need to maintain structural integrity, mechanical compatibility with host tissues, and stability under physiological temperature fluctuations—all while meeting the stringent safety and functional requirements necessary for long-term implantation within the human body. Especially in biocompatible thermoplastics, cyclic humidity conditions aggravate phenomena like moisture-accelerated creep. Creep is the time-dependent, permanent deformation of a material when it is subjected to a constant load or stress over time, even if that load is below the material’s yield strength and is influenced by the body temperature (~37 °C), the moist environment, and the cyclic or continuous mechanical loading [109]. The creep phenomenon may lead to loss of structural integrity (e.g., spinal cages, orthopedic plates); the loosening, deformation, or failure of prostheses; geometric and dimensional changes in scaffolds; or fatigue damage acceleration.

A great advantage related to the mechanical testing of biomaterials is that, compared to chemical and the biological characterizations, it benefits from clear guidelines and standards that are already described in detail and that were developed for materials with applications in several industries. In the category of static mechanical characterization, the commonly employed standards are available at [110,111]:

(a) Tensile testing to determine the tensile strength, elastic modulus, and elongation at break in the case of polymers, metals, and soft materials is performed according to ASTM D638 (polymers), ASTM E8 (metals), or other standards for micro-tensile testing when samples are small or very thin (ASTM D1708 etc.).
(b) Compression tests to evaluate compressive strength and modulus, applied in the case of synthetic bone materials, scaffolds, and hydrogels according to ASTM D695.
(c) Flexural tests (usually three-point bending and sometimes four-point bending) for measurement of flexural strength and modulus in ceramics, composites, and scaffolds (ASTM D790).
(d) Fracture toughness tests for resistance to crack propagation in brittle materials like ceramics (ASTM E399, ISO 23146).
(e) Creep tests to determine time-dependent deformation under constant load in polymers and gels (ASTM D2990).
(f) Stress relaxation tests, which consist of a decrease in stress under constant strain in polymers (ASTM E328, ASTM D7015, ISO 16770).

Cyclic loading is performed using some of the above tests for the assessment of the fatigue resistance and the degradation over time (aging) of biomaterials. The evaluation that results from the static experiments is used in the design and programming of the properties for adequate orthopedic materials, scaffolds, hydrogels, synthetic soft tissues, and dental materials. While the mechanical properties discussed so far are typically evaluated under static conditions or at low strain rates (i.e., low crosshead speeds), real-life scenarios expose implanted biomaterials to high-strain-rate conditions. In such dynamic events, the material’s behavior can differ significantly, and mechanical properties obtained from static tests are not reliable predictors of fracture performance. To accurately assess this behavior, it is essential to measure the strain-rate-dependent dynamic strength and to understand how damage mechanisms evolve throughout the course of dynamic loading. This is especially relevant for brittle ceramics, where dynamic fracture can occur within a few hundred microseconds, in contrast to the much longer timescales observed in static tests. Therefore, evaluating the dynamic strength of biomaterials is crucial for ensuring their reliability under sudden or extreme mechanical loads [112]. Moreover, thermomechanical analysis used to observe the transition temperature of thermoplastics from glass (crystal) to a rubbery state, and the threshold where its thermoelastic behavior is over and permanent plasticization occurs, is necessary. As previously mentioned, the changes in properties might not be significant when a thermoplastic performs in the body microenvironment for up to a few weeks, but damage can suddenly accelerate after the resistance threshold ends.

Since biomimetic body-like environment testing is recommended, Dynamic Mechanical Analysis (DMA) must be applied to understand the biomaterial’s dynamic behavior, including its viscoelastic and dynamic response when under thermal and dynamic loading in a relevant environment. Moreover, a low impact is relevant if the implant is placed in the upper layer of the skin or at a body joint. Depending on the activity of a human (e.g., driving or a specific job-associated activity), tangential external forces impact the implant and its functionality.

Some other important tests for accurate material characterization in a relevant environment are the following:

Measurements of the Melt Flow Index (MFI, ASTM D1238): Any thermoplastic material’s MFI is determined by passing it through a melt flow tester for 10 min under specific standard load and temperature conditions. The flowability of thermoplastic materials is indicated by the MFI. A device known as a melt flow tester is used to verify this well-known melting characteristic of thermoplastic materials. This test is particularly important in the case of hydrogels.

Differential scanning calorimetry (DSC, ASTM D3418, ISO 11357 series, ISO 6721-11): Measures how a biomaterial’s heat flow changes with temperature, revealing key transitions like glass transition (T_g_), melting point (Tm), and crystallization, which are critical for understanding its stability, processing, and performance in biological environments.

Dynamic Mechanical Analysis (DMA) is used to investigate the viscoelastic behavior of thermoplastic biomaterials. Using rectangular specimens, ASTM D5026 standards are used to determine the storage (elastic or E′), loss (viscous or E″), and complex (E*) moduli, as well as tan delta (δ), as a function of frequency, temperature, or time. These properties provide insights into the thermomechanical performance, including the glass transition temperature (T_g_), from glass to a rubbery state, and the damping behavior. Especially in biomaterials designed for bone application, poor damping will result in a transfer of shocks to bone, leading to pain, implant loosening, and early failure. On the other hand, thermoplastics with controlled damping absorb energy and reduce wear on surrounding bone and tissues. This method was successfully applied by researchers to measure the properties of difficult materials like hydrogels [113].

Low-energy impact testing (ASTM F1839, ASTM F1044, etc.) is used in biomaterial testing to evaluate the resistance to microcracks or surface damage, mimic physiological loads, assess fracture toughness in brittle materials, and determine suitability for load-bearing applications. Low-energy impact testing simulates realistic, sub-catastrophic forces that can affect the function, safety, and durability of medical devices, especially in the outer casing of electronic devices. The box that will contain the non-compatible inner elements like cables and electrolytes must be subjected to low-energy impact testing.

Thermogravimetric analysis (TGA, ASTM E1131, ASTM D3850, ISO 11358-1 to 3, and others) is a technique used to measure the mass change of a material as a function of temperature or time under a controlled atmosphere. It provides information on the decomposition temperature and amount of remaining inorganic residue, which is important for the degradation of biomaterials and the potential toxic effect, especially in thermoplastics; and thermal stability, which is also important for long term biomaterial performance in the relevant environment. It is also successfully applied in polymer blend or composite analysis. TGA, therefore, is important for understanding processing and end-use properties, especially in applications like biodegradable packaging or biomedical devices.

Since composite materials offer mechanical support to biological tissues and exhibit biocompatibility and non-toxicity, facilitating their manipulation into intricate structures and shapes, their application in biomedicine is attracting more attention than ever. The above experimental approaches, tools, and devices are also used in the assessment of the performance of composite biomaterials. However, it is important to mention that in the case of composite materials, a thorough imaging investigation to observe the situation at the contact between the two or more material phases that form the composite, and other supplementary tests (i.e., delamination for multilayered composites), are required.

#### 3.2.4. Biological Characterization

Biocompatibility testing with cell cultures is a later step in testing a biomaterial’s feasibility, after performing its surface, chemical, and mechanical characterization in a relevant environment. This is because although a biomaterial might positively trigger the cell culture in vitro for a determined period of up to one month, the in vivo response is different if the material is not chemically and mechanically reliable. Biocompatibility is several times confused with “cell culture testing”, while this is one of the last characterization steps. The characterization of the biomaterial must successfully pass the chemical tests to prove non-toxicity, as mentioned in Section 3.2.2, followed by testing for structural integrity (mechanical testing) and afterwards by biological testing. In biocompatibility testing, e.g., ISO 10993-5, primary cells directly isolated from human tissues are preferred for advanced, tissue-specific testing because they are physiologically relevant and more adequate for predicting the in vivo responses since they have no artificial adaptation mechanisms. However, before starting the tests with cell cultures, the hemocompatibility characterization must be performed.

In Section 3.2.2, we mentioned that the implantation of a biomaterial enables chain reactions at the insertion sites. The main events are mentioned in Table 1. 

In the first seconds to minutes, blood covers the surface of the biomaterial and specialized receptors set off the events through a cumulative effect. Protein adsorption is the first step in the interaction of the biological components and the biomaterial. This step can be investigated in vitro by surface and chemical analysis of protein adsorption at the biomaterial surface, after its exposure to cell culture medium, through methods that were presented in Section 3.2.1 and Section 3.2.2. Later, there is a platelet activation that indicates the “awakening” of the immune system. In vitro, hemocompatibility testing with platelets obtained from human donors is the most adequate. A protocol is described below. A more aggressive response from the immune response can be investigated in vitro with neutrophils and macrophage cell cultures [114]. Angiogenesis expresses the vascularization capacity of a novel biomaterial and can be investigated with models like endothelial cell tube formation assay for angiogenesis [115]. Finally, in the events cascade in Table 1 we observe that fibrosis is the last potential manifestation of implant rejection. It consists of the isolation of the biomaterial/biodevice through encapsulation, which happens generally when there is no biointegration and the material is inert, leading to non-recognition. The impact of biophysical factors on fibrosis has been partially understood. To begin to understand forces generated within tissues, in vitro self-assembled microtissues have been developed. Early in vitro studies of fibrosis were primarily conducted on rigid substrates like tissue culture polystyrene (TCPS) or glass, which promote a fibrotic cell phenotype even in the absence of external signaling cues. To overcome some limitations, advanced engineered systems have been further developed that allow for 3D culture for fibrosis investigation [116].

The ISO 10993-5:2009 standard “Biological evaluation of medical devices Part 5: Tests for in vitro cytotoxicity” describes the protocol for cytotoxicity testing of biomaterials. Mutagenicity and genotoxicity tests must be performed according to the ISO 10993-3:2014 standard “Biological evaluation of medical devices Part 3: Tests for genotoxicity, carcinogenicity and reproductive toxicity”. For these experiments, the recommended cells are CHO-K1 cell line (Chinese hamster cells), and they involve, among other things, good growth and a stable karyotype [117].

Between the above tests and regarding biointegration, the most powerful tools use primary human cells of different types for the design of application correlated experiments (e.g., dermal cells to test food packaging materials, osteoblasts to evaluate bone synthetic materials, chondrocytes for cartilage, etc.). Furthermore, it has to be mentioned that for the biointegration concept that aims at focusing on the biomaterial–cell interphase and its associated processes, biocompatibility testing must be performed with adherent cells like stem cells, osteoblasts, and fibroblasts, which stick on the surfaces and immediately respond to the microenvironment by modulating their morphology, population size, distance between them, and other aspects. The challenge in this direction consists of the fact that the standards do not detail a protocol with clear investigation steps for the deposition and monitoring of the different physiological cell cultures on the biomaterials. In this case, bibliographic studies are not comparable with each other and a significant scientific input is lost. A protocol that combines literature knowledge and personal experience is proposed by the authors that underlines some basic steps in biomaterial biological assessment.

Hemocompatibility assessment

**Figure 5:**
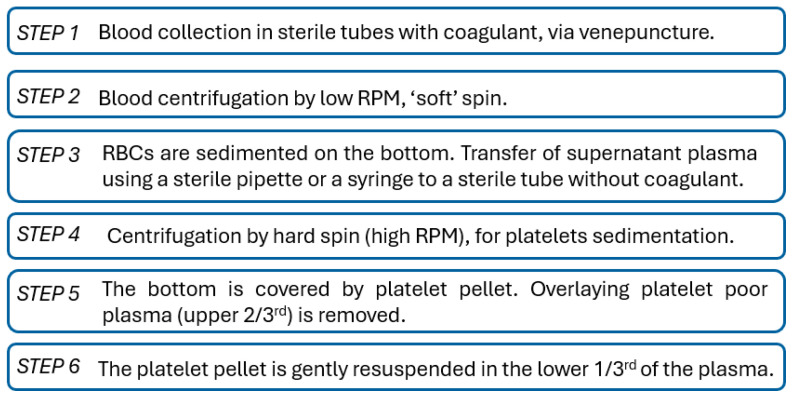
Step-by-step method of preparation of PRP using the double-spin open method, as described by Dashore S et al. [118].

Platelet-Rich Plasma (PRP) is a blood derivative with a high concentration of platelets and associated growth factors and cytokines commonly used in regenerative medicine, which also serves in biomaterial evaluation to evaluate platelet adhesion, activation markers, morphological changes, and thrombus formation potential. Because there was a lack of standardization of PRP preparation in the medical literature, Dashore S et al. [118] investigated and reported the methodology of PRP preparation. The main steps are described in Figure 5. According to numerous studies and the technical manual of the American Association of Blood Banks, double spin is the accepted technique for preparing PRP. The heavier red blood cells (RBCs) are the first spin sediments. After being moved to a different tube, the liquid supernatant is centrifuged again at higher RPM. After discarding the top two-thirds of the cell-free supernatant, the cell pellets are resuspended in a smaller volume of plasma.

The obtained PRP must be seeded on the tested substrates. Hemocompatibility is assessed through observation of the platelets’ distribution on the substrates: platelets’ number, density, dimensions, distribution, etc.

Key aspects in biocompatibility testing with primary adherent cell cultures

The literature lacks a description of the technical laboratory steps used in evaluating biocompatibility with adherent cells in relation to a substrate. There are some crucial aspects when starting such a study, considering that a common experimental design will allow the comparison of the results with other bibliographic studies:

The studies must be performed against TCP (tissue culture polystyrene), which is the standard material for in vitro maintenance of cell populations. The results on the novel biomaterials must be compared with those of the TCP. To ensure that this is possible, all the surface areas of the materials must be of the same dimensions. However, in the case of 3D scaffolds, since it is difficult to obtain 3D scaffolds from TCP, the study can be performed by analogy.

Two important aspects of cells’ deposition must be considered: to use a Neubauer chamber in order to ensure the deposition of the same cell number/sample, cell density must be adjusted with a Neubauer chamber to have a specific number of cells in an exact quantity of cell culture medium that will be delivered to all the samples, and to ensure that the cells are deposited in the center and on different points of the sample equidistant from its center.

The first key indicator of cells’ response to a surface is their proliferation rate, which refers to more than the multiplication. Proliferation can be defined as the multiplication corelated to surface area, in a specific given time.

Morphologic development is one of the key indicators of biocompatibility. In addition to an adequate shape, cells must have also appropriate dimensions. For this, radial measurement or length and width measurements must be performed [119].

The migration on the surface area and the cells’ network formation is important. This can be appreciated by measuring distances between cells in the cell population, on the various tested substrates. All the data will contribute to the calculation of the rate of in vitro tissue formation.

The attachment to the surface resulting from focal adhesion point analysis, in relation to the total protein levels [102] and correlated with all the above aspects, will offer exact and valuable information on the cell population’s preferences towards substrates.

The most important biomarkers specific to each cell type must be investigated and whenever possible, corelated between each other.

When the experimental setup allows it, the above investigation becomes more valuable if performed in a relevant physiological environment like, for example, a bioreactor (Section 4). When testing powders, medicine, or hydrogels, an adapted protocol is recommended by administration of various doses to the cell culture, via the cell culture medium, and subsequent identification of the following thresholds vs. time: (1) multiplication stop followed by necrosis in the case of long-term exposure; (2) dysfunctionality resulting in necrosis after moderate-term exposure; or (3) necrosis/apoptosis—instant death.

As mentioned previously, specific limitations exist in ISO standards regarding the testing of innovative classes of biomaterials, which are nanomaterials and 3D scaffolds. While the engineering tools and methods are accurately described, biocompatibility testing when these materials meet the biological component results in several challenges. In this direction, the authors propose some laboratory solutions:

1. Nanomaterials can be tested in contact with human cell cultures as follows: the electrostatic nanomaterials (e.g., carbon-based materials) can be deposited on a biocompatible semiconductive plate like titanium as a coating. Further, cells can be deposited and monitored on the nanomaterial coating. A second solution for non-electrostatic materials is the administration of various doses of nanomaterial in the cell culture medium, as in the case of powders, pharmaceuticals, or hydrogels, and the monitoring of cells response as described above.
2. The challenge with 3D scaffolds is related to the deposition of the cells. Regarding the number of cells and their monitoring, the above-described steps for traditional material testing can be followed. However, an important detail of the deposition is to maintain the drop of cell culture medium with cells in several points of the scaffold fiber, while in an incubator, for up to three hours to enable adhesion. Afterwards, scaffolds with cells can be totally immersed in cell culture medium. Following this protocol, cells will not be lost in the pores and they will be maintained on the scaffold. The imaging of the cells must be performed while they stay on the scaffold. However, biomarker analysis can be performed after removing the cells from the scaffold.

## 4. Optimization of Experimental Setup, Customized for Relevant Testing

Because food packaging materials belong to one of the most developed industries worldwide, testing standards are well defined and described in detail. Firstly, the food packaging material must be biocompatible—non-toxic and non-allergenic—when in contact with the skin. Later, the mechanical performance of the food packaging materials is at the center of the experimental characterization. The testing modes are the tensile and compression tests, complemented by drop and vibration tests. Another important test specific to food packaging materials is the test of heat seal integrity. The strength and efficiency of sealing the packaged goods affect the predicted shelf-life of food products. The sealing should be proper and it should hold the material inside without any leakage. Physical characterization includes specific tests, like water vapor transmission and the relative humidity sensor, which allow testing in a relevant environment. In these methods, wet and dry chambers are maintained at high and low relative humidity, respectively. This allows the study of the transfer of moisture from a package into the environment [14]. In appropriate studies, environmental chambers, incubators, or customized containers are used to mimic the environmental conditions to which food packaging materials are exposed, including temperature oscillations, humidity, and gases [14].

In the case of implantable biomaterials and biodevices, the situation is more complicated. The elaboration of complex laboratory systems that allow the partial replacement of animal model experimentation with advanced in vitro bioreactor-based models is currently the target. This is in line with Directive 2010/63/EU for the development and promotion of non-animal approaches to testing and research. Among its tasks is the validation of methods for the 3Rs—replace, reduce, or refine the use of animals for the safety and efficacy/potency testing of chemicals, biologicals, and vaccines. Validating innovative methods and protocols is necessary to ensure biomaterials and biodevices meet the rigorous standards required by regulatory bodies, facilitating their acceptance and integration into regulatory frameworks.

To prepare a setup that biomimics the body’s conditions, a dynamic experiment must be applied, where the body fluids and the blood flow [120] are important factors influencing biomaterial degradation. On the other hand, blood components (red and white blood cells, platelets, water, salts, and proteins) do not directly impact the implanted biomaterial. Blood velocity creates pressure and vibrations that indirectly affect the biomaterial. Biofluids such as electrolytes, solutes, and other molecules (enzymes, amino acids, cells, etc.) dissolved in aqueous medium meet the biomaterial and flow around and through it. The flow rate Q is defined to be the volume of fluid passing by some location through an area during a period. The situation is complex and the in vitro biomimicking of the microenvironment is an ambitious task. However, by introducing key parameters such as body temperature accompanied by dynamic flow of the cell culture medium, a more accurate approach to reality may be achieved.

The replacement of the salt-based simulated body fluids with a cell culture medium in the experiments is a gain even in the absence of a cell population, since the tested biomaterial will meet important biological components like amino acids, enzymes, protein, and blood cells, enabling processes mimicking the in vivo microenvironment. The components of a commonly used cell culture medium are given in Table 2.

The temperature must be regularly adjusted with a thermometer, and the constant pH of the cell culture medium must be checked as well. The amount of cell culture medium should be maintained constant, as well as the initial pH value. For experiments performed with a magnetic stirrer, to obtain a dynamic rotation of the samples and to approach the average blood flow velocity at rest in the capillaries an angular frequency of 300 rpm can be applied for the experiment.

**Figure 6:**
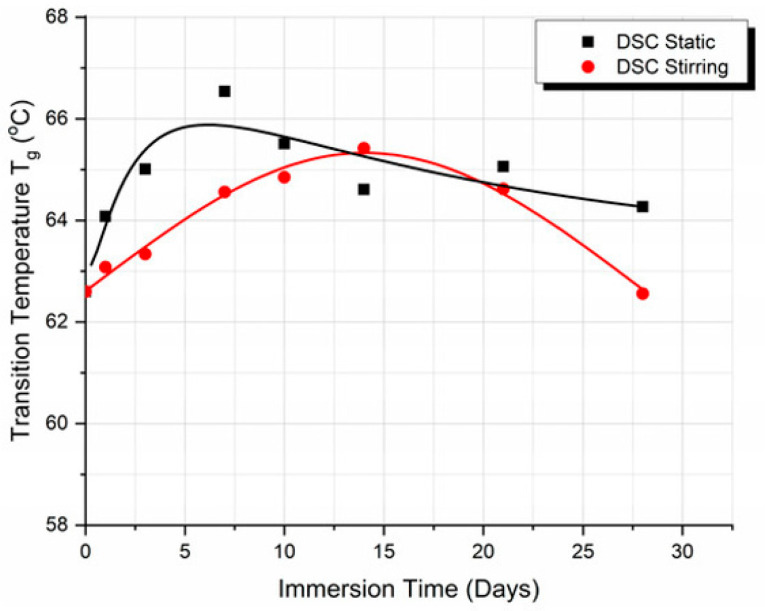
Glass transition temperature (T_g_) of 3D-printed PLA scaffolds—variation with immersion time when under static and stirring conditions [120].

A previous investigation was conducted to observe the importance of the effect of a dynamic microenvironment on the degradation profile of 3D-printed PLA scaffolds [121]. The designed experiments consisted of immersing the samples in culture media for up to one month in static and dynamic conditions. The body temperature was maintained constant. Clear differences were found between the effects of the two types of experiments. According to the SEM examination, the samples from the stirring experiment are more brittle, whereas the samples from the static experiment are more damaged. The material’s maximum T_g_ value, as determined by DSC, was around 65 °C. This value was attained after five days of static immersion and fourteen days of stirring immersion (Figure 6), suggesting that certain processes take place more quickly in the static experiment.

Another study aimed at the reproduction of a biomimetic environment for human cell cultures by applying an electrical current to simulate the natural cells’ bioelectricity [45]. An electrical stimulation device has been manufactured to allow the maintenance of human cells in an incubator under electric current. The homogeneous electric field distribution induced by a highly organized titanium dioxide nanotube substrate had an optimum effect on cell response. Specific substrate topography in combination with appropriate electrical stimulation enhanced osteoblast bone cells’ capacity to self-adjust the levels of their specific biomarkers. Since the human body is a natural electrochemical cell, the application of the electric field is one of the parameters that can improve the quality of the in vitro measurement, reflecting situations closer to the physiological conditions. In another study on synthetic bone scaffolds, which involved human cells, the cell culture medium was used under flow conditions in a bioreactor. In this way, the dynamic environment of the body was partially reproduced and a more realistic investigation to compare the performance of different scaffolds was achieved [122].

In another study [123], hydrogel scaffolds made of porous polysaccharides were paired with a perfusion device similar to a bioreactor, which improves nutrient transfers and creates advantageous mechanical stresses. Mouse osteoblasts were seeded and grown for three weeks in dynamic circumstances. It was discovered that whereas cell viability declined in the static culture, it remained constant in the dynamic circumstances. The presence of an ideal spheroid size and spacing for proper oxygenation is suggested by the simulation of oxygen transport and consumption in the bioreactor, which verified that the cells in spheroids did not experience hypoxia when the bioreactor was perfused. These results made it possible to identify the ideal bioreactor conditions for effective in vitro cell organization and spheroidal survival, which are critical for future organoid applications.

A very complex study [124] in a bioreactor allowed the understanding of the niche interactions between blood and bone through the in vitro co-culture of osteo-competent cells and endothelial cells. To facilitate the spontaneous development of spheroids, a mesenchymal stem cell line and a human umbilical vein endothelial cell line were co-cultured under flow conditions in a three-dimensional, porous, natural pullulan/dextran scaffold that was enhanced with hydroxyapatite crystals. After two weeks, dead cells were centered in the spheroids, and their viability was higher under dynamic settings (>94%) than static conditions (<75%). Under the dynamic conditions, mineralization and collagen IV production were enhanced in tandem with angiogenesis and osteogenesis. By day seven, the endothelial cells had gathered at the spheroidal core. In the dynamic settings, proliferation doubled, particularly along the edges of the scaffold.

Overall, the application of a complex dynamic environment in biological testing of biodevices reflects a response closer to reality from the cell/tissue side. However, at the moment there are no metrics to define the efficiency for bioreactor-simulated environments compared to physiological conditions, since it is impossible to reproduce all the factors in the body. In this case, researchers may focus on reproducing the key conditions: mechanical stimulation for orthopedic implants, electricity for nervous synthetic tissues, elasticity for dermal applications, etc., which allow a more mature interpretation of the results. Finally, the bioreactor conditions partially mimic the physiological environment and allow an adequate evaluation of the performance of biomaterials, as well as appropriate conditions for improved cell populations functioning in vitro.

## 5. Conclusions

This research presents an overview of the challenges encountered in the relevant testing of innovative biomaterials and implantable medical devices. The primary objective is to establish a correlation between the design of biomaterials or biodevices and their operating environments, with the aim of enhancing performance optimization. In this context, biomaterials are defined as any material that comes into direct contact with the human body, including food packaging materials, conventional implant materials, and complex implantable medical devices.

Given the multidisciplinary nature of the field, a comprehensive understanding of principles across biology, engineering, materials science, and medicine is essential. Identifying the most influential parameters affecting biomaterials necessitates close collaboration among experts from these disciplines.

Since replicating the exact conditions of a bioproduct’s operating environment in laboratory settings is nearly impossible, it is crucial to identify and simulate the key environmental factors that influence performance and degradation. Food packaging materials are exposed to fluctuating temperatures, varying humidity, and mechanical stress. Some of them are not required to perform in fluidic environments. Nonetheless, some might be subjected to fluidic environment by intention or accidentally, while their exposure to external elements as a broader range of microbial and viral contamination than biomedical materials is significantly higher.

Effective testing begins with defining the microenvironmental conditions and tailoring biomaterial properties to meet functional requirements. To streamline development and reduce reliance on trial-and-error approaches, the authors propose a strategic framework to minimize time and cost during bioproduct development. The key steps are as follows:

1. Application-oriented design and fabrication: This involves leveraging computational and analytical modeling, alongside advanced engineering tools, to develop structures optimized for specific applications.
2. Selection of relevant standards: Identifying and adopting appropriate standards aids in defining testing protocols. This facilitates partial accreditation and ensures comparability with existing bioproducts.
3. Development of customized testing protocols: Beyond standard procedures, advanced and application-specific testing methods using customized devices should be implemented.

The authors advocate for a “biointegration” approach, which involves the design of biomimetic materials that support biorecognition. Biointegration goes beyond mere biocompatibility, aiming for a complex interaction between the biomaterial and host tissue to promote optimal integration with minimal or no adverse effects. This approach necessitates pairing conventional testing with relevant environmental simulations to achieve comprehensive material characterization.

Traditional biomaterial evaluation methods must be adapted to more sophisticated and context-aware protocols. This adaptation should begin with material classification:

- Metallic materials should be assessed for corrosion resistance. Techniques such as cyclic voltammetry can be used to monitor ion release over prolonged exposure in simulated body fluids and develop corresponding mitigation strategies.
- Carbon-based materials, though generally considered non-toxic, encompass a wide variety that requires individual toxicity and long-term stability assessments, particularly regarding their electrical properties after long exposure to the relevant environment.
- Thermoplastics must be tested for viscoelastic behavior, particularly under cyclic temperature fluctuations or complex mechanical loads, as encountered in both environmental (e.g., packaging) and biomedical (e.g., implants) applications.

Comprehensive characterization should begin with analysis of structural and chemical properties, focusing on the potential release of ions, macromolecules, and other byproducts that may impact human health. Mechanical performance assessment follows, which can be particularly challenging due to the inherent fragility of many biomaterials. Only materials that meet both chemical and mechanical criteria should proceed with biological testing.

All testing should ideally occur after conditioning the biomaterials or biodevices in laboratory settings that closely replicate natural environments. Tools such as bioreactors, specialized containers, and incubators are essential for simulating consistent or variable environmental conditions. For implantable materials, simulated body fluids enriched with complex biological elements (e.g., cell culture media) are preferable. Additionally, dynamic conditions—including flow, mechanical stress, rotation, and electric fields—should be incorporated to ensure relevance. It is acknowledged that in vitro bioreactor-based research may still present limitations—such as its inability to capture systemic biological responses (e.g., immune cascades, chronic inflammation)—because it may not replicate multi-stimuli systems. However, the sequential investigation of biological events provides valuable insight into the pairing of natural tissues with synthetic medical bioproducts, opening new perspectives in food-related products, tissue engineering, and artificial organs. Moreover, the framework of the present investigation—imaging, chemical testing, mechanical testing, and finally biological assessment—can be adapted for emerging biomaterial types not covered in this review. The goal is to harmonize research methodologies by establishing standardized experimental conditions. This will facilitate data comparability across fabrication methods, material performance assessments, and the evaluation and accreditation of testing effectiveness in a relevant microenvironment.

## Figures and Tables

**Table 1:** The duration of different events that are enabled post implantation.

Event duration	Physiological activity.
Seconds–Minutes	Protein adsorption (largely determines subsequent cell responses).
Minutes–Hours	Platelet activation, clotting (activation of coagulation and complement cascades; hemocompatibility must be tested).
Hours–Days	Neutrophil recruitment (acute inflammation).
Days–Weeks	Macrophages (chronic inflammation).
1–4 Weeks	Granulation tissue, angiogenesis (new blood vessels form angiogenesis to support healing).
Weeks–Months	Fibrous encapsulation or integration (a fibrous capsule forms, isolating the implant; this is often seen with an inert or non-degradable implant).

**Table 2:** Composition of the cell culture medium used for the biodegradation examination.

Substances	Composition	Function
α-MEM	169 mL	Basal growth medium
Fetal bovine serum	20 mL	Hormone factors for proliferation and growth
Amphotericin B	2 mL	Antifungal supplement
L glutamine	2 mL	Supplement for protein synthesis
L ascorbic acid	2 mL	Antioxidant, i.e., to suppress reactive oxygen species generation
Penicillin–streptomycin	1 mL	Antibiotics for Gram-positive and Gram-negative
